# A Smart Card-Based Electronic School Absenteeism System for Influenza-Like Illness Surveillance in Hong Kong: Design, Implementation, and Feasibility Assessment

**DOI:** 10.2196/publichealth.6810

**Published:** 2017-10-06

**Authors:** Dennis KM Ip, Eric HY Lau, Hau Chi So, Jingyi Xiao, Chi Kin Lam, Vicky J Fang, Yat Hung Tam, Gabriel M Leung, Benjamin J Cowling

**Affiliations:** ^1^ WHO Collaborating Centre for Infectious Disease Epidemiology and Control School of Public Health, Li Ka Shing Faculty of Medicine The University of Hong Kong Hong Kong China (Hong Kong)

**Keywords:** influenza, public health surveillance, school health, absenteeism, smart cards

## Abstract

**Background:**

School-aged children have the highest incidence of respiratory virus infections each year, and transmission of respiratory viruses such as influenza virus can be a major concern in school settings. School absenteeism data have been employed as a component of influenza surveillance systems in some locations. Data timeliness and system acceptance remain as key determinants affecting the usefulness of a prospective surveillance system.

**Objective:**

The aim of this study was to assess the feasibility of implementing an electronic school absenteeism surveillance system using smart card–based technology for influenza-like illness (ILI) surveillance among a representative network of local primary and secondary schools in Hong Kong.

**Methods:**

We designed and implemented a surveillance system according to the Protocol for a Standardized information infrastructure for Pandemic and Emerging infectious disease Response (PROSPER). We employed an existing smart card–based education and school administration platform for data capture, customized the user interface, and used additional back end systems built for other downstream surveillance steps. We invited local schools to participate and collected absenteeism data by the implemented system. We compared temporal trend of the absenteeism data with data from existing community sentinel and laboratory surveillance data.

**Results:**

We designed and implemented an ILI surveillance system utilizing smart card–based attendance tracking approach for data capture. We implemented the surveillance system in a total of 107 schools (including 66 primary schools and 41 secondary schools), covering a total of 75,052 children. The system successfully captured information on absences for 2 consecutive academic years (2012-2013 and 2013-2014). The absenteeism data we collected from the system reflected ILI activity in the community, with an upsurge in disease activity detected up to 1 to 2 weeks preceding other existing surveillance systems.

**Conclusions:**

We designed and implemented a novel smart card technology–based school absenteeism surveillance system. Our study demonstrated the feasibility of building a large-scale surveillance system riding on a routinely adopted data collection approach and the use of simple system enhancement to minimize workload implication and enhance system acceptability. Data from this system have potential value in supplementing existing sentinel influenza surveillance for situational awareness of influenza activity in the community.

## Introduction

### School as a High-Risk Setting for Disease Transmission

Transmission of respiratory virus infections, including influenza virus infections, has always been a major concern in schools because of poorer personal hygiene practices, more frequent person-to-person close contacts, and relatively lower preexisting immunity among young children [1,2]. Besides associated morbidity and mortality, respiratory virus infections can also result in disruption to learning because of sickness absence [3] or school closure [4]. Outbreaks of influenza-like illnesses (ILIs) in schools often precede and may lead to further community epidemics, with transmission through family members [5].

### Surveillance of School Absenteeism

School absenteeism is now increasingly being employed as a source of syndromic data for influenza surveillance to inform epidemic and pandemic preparedness in different locations [6-8]. Whereas previous studies generally suggested that school absenteeism data is useful for reflecting community influenza activity [9-11] and detecting outbreaks [12,13], data timeliness and system acceptance remain as key determinants affecting their usefulness for prospectively informing relevant public health actions and reducing disease transmission [14,15].

### Use of Smart Card Technology for Disease Surveillance

Although verbal roll call with paper-based records still represents the predominant approach for attendance taking in most schools all over the world, a number of newer electronic means of attendance tracking, including the use of smart card technology, have also emerged in the market. A smart card (also called chip card or integrated circuit card) is a pocket-sized plastic card embedded with integrated circuits, allowing for individual identification, security authentication, data storage, and application processing in a wide variety of settings, including banking, retail business, bill payment, transportation, custom control, and educational settings [16]. In recent years, smart card technology–based systems are increasingly being adopted for various administration purposes in all levels of local educational institutions. In this respect, the situation in Hong Kong offers an unprecedented opportunity for assessing whether these automated data collection approaches in a school setting may offer the potential to contribute to more timely and efficient disease surveillance. In a previous pilot study, we demonstrated the feasibility of employing smart card–based technology to capture school absenteeism data for surveillance purposes [17]. Here, we report further details on the implementation and feasibility of building a comprehensive system for prospective ILI surveillance among a representative network of local primary and secondary schools in Hong Kong. On the basis of smart card technology, the system was being utilized to track absence from school, supplemented with additional back end systems for the automation of all subsequent steps required in the surveillance process, including a process to capture the reasons for absence.

## Methods

### Scientific Methodological Approach

We designed and implemented our surveillance system according to the standardized information infrastructure as laid out in the Protocol for a Standardized information infrastructure for Pandemic and Emerging infectious disease Response (PROSPER), with a view to better inform the three specific functions in relation to supporting influenza pandemic responses, namely capacity and needs analysis (CNA), response design modeling (RDM), and outcome and impact assessment (OIA) functions [18].

### Study Setting

Currently, ILI surveillance in Hong Kong is administered under the Centre for Health Protection (CHP) of the Department of Health. The CHP prospectively monitors a number of data streams from different networks of surveillance partners covering different population subsectors. Sentinel doctors’ networks included some 50 general practitioners (GPs) in private practice, 65 general outpatient clinics, and sentinel Chinese medicine practitioners in selected clinics, each reporting the proportions of patient consultations having ILI (a body temperature of 38.0°C plus cough or sore throat) on a weekly basis. Other additional systems included child care centers and kindergartens reporting children suffering from fever or cough and residential care homes for the elderly reporting inmates having fever [19]. For primary and secondary schools in Hong Kong, however, a mechanism does not exist for the prospective monitoring of ILIs besides the passive reporting of any confirmed or suspected outbreaks. After the severe acute respiratory syndrome epidemic, most schools in Hong Kong have required parents to monitor body temperature of their children every day and to abstain from sending them to school when having a fever, making absenteeism a suitable proxy for disease surveillance [20]. Many schools in Hong Kong now use smart cards rather than verbal roll call to track attendance at school because of convenience and efficiency.

### Design and Implementation of the Surveillance System

In this study, we collaborated with BroadLearning Education (Asia) Limited, the commercial developer of a Web-based education and school administration platform *(eClass)* and employed its smart card–based attendance tracking functionality *(eAttendance)* for frontline data capturing. The *eClass* is an integrated platform for learning, administration, and communication, and *eAttendance* is a module that allows automatic generation of attendance report for internal use, as well as in a format ready for submission to the Education Bureau in Hong Kong. In a pilot study run from 2008 to 2011, captured data were sent to us from 20 schools by email on a daily basis. In this study, we used the same approach for frontend automatic data capture, with additional back end components developed, using scripts in the Linux Web server, to automate all subsequent steps in the surveillance process, including data transfer, cleaning, aggregation, analysis, and feedback intelligence report generation. The original user interface of the school administration system was customized with additional specific features aiming to improve data specificity and completeness and to minimize the time and workload involved in data submission. This study was approved by the institutional review board of the University of Hong Kong (HKU)/Hospital Authority Hong Kong West Cluster.

### Recruitment of Schools

We extended our initial invitation to all local schools using the *eAttendance* system, targeting to recruit around 10.00% of local schools to participate in the surveillance system. Interested schools were visited by our research team to provide further explanation on study details and logistical arrangement. In consenting schools, we updated their *eClass* system as described in the following sections.

### Data Collection

After implementing the system, absenteeism data were prospectively collected daily from all participating schools for 2 academic years using the system. We obtained data on community sentinel ILI surveillance from the CHP website and laboratory surveillance data from a regional tertiary hospital in Hong Kong (Queen Mary Hospital) as reference data and compared their temporal pattern with that of the absenteeism data. We also obtained feedback from participating schools regarding their experience of using the system by a short questionnaire at the end of the study.

## Results

### Technical Details of the Implemented Surveillance System

**Figure 1:**
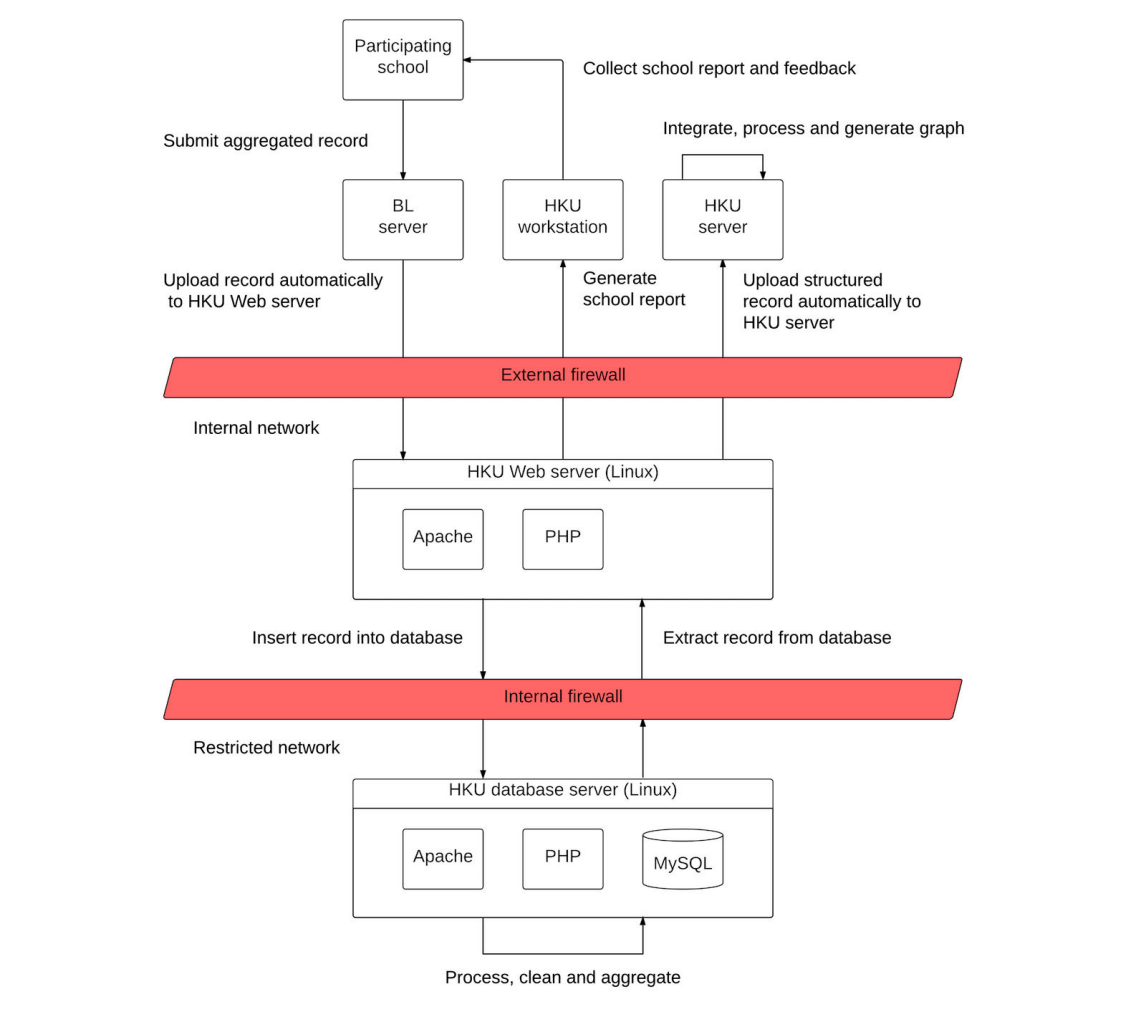
System architecture of the smart card-based school absenteeism surveillance system. BL: BroadLearning, HKU: The University of Hong Kong, PHP: Hypertext Preprocessor, MySQL: structured query language.

All system design and information technology implementation works were completed by May 2012. Figure 1 shows the overall system and data flow architecture of the school absenteeism surveillance system.

### Data Capture

**Figure 2:**
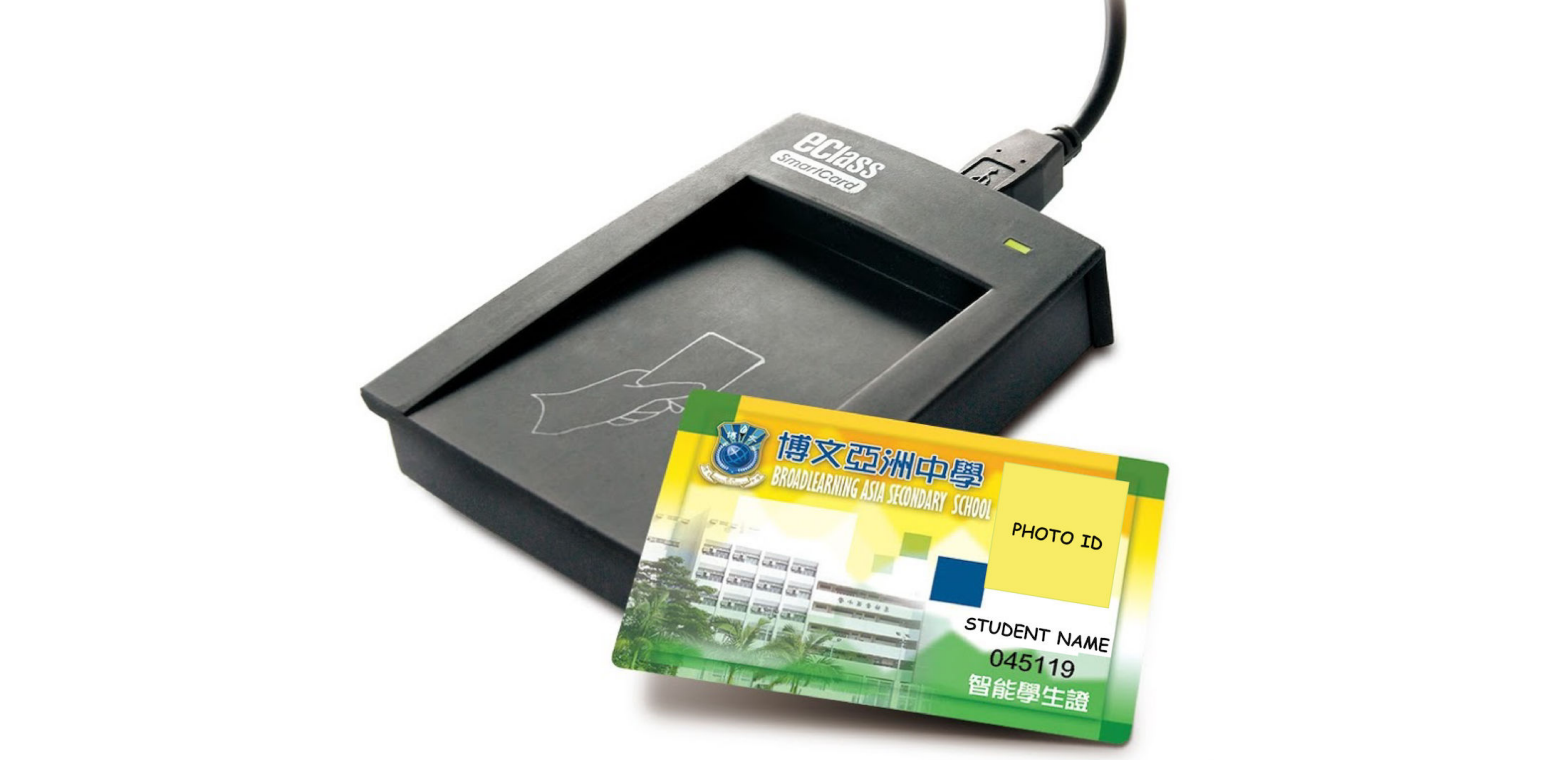
Example student identity card (anonymized) and smart card reader.

**Figure 3:**
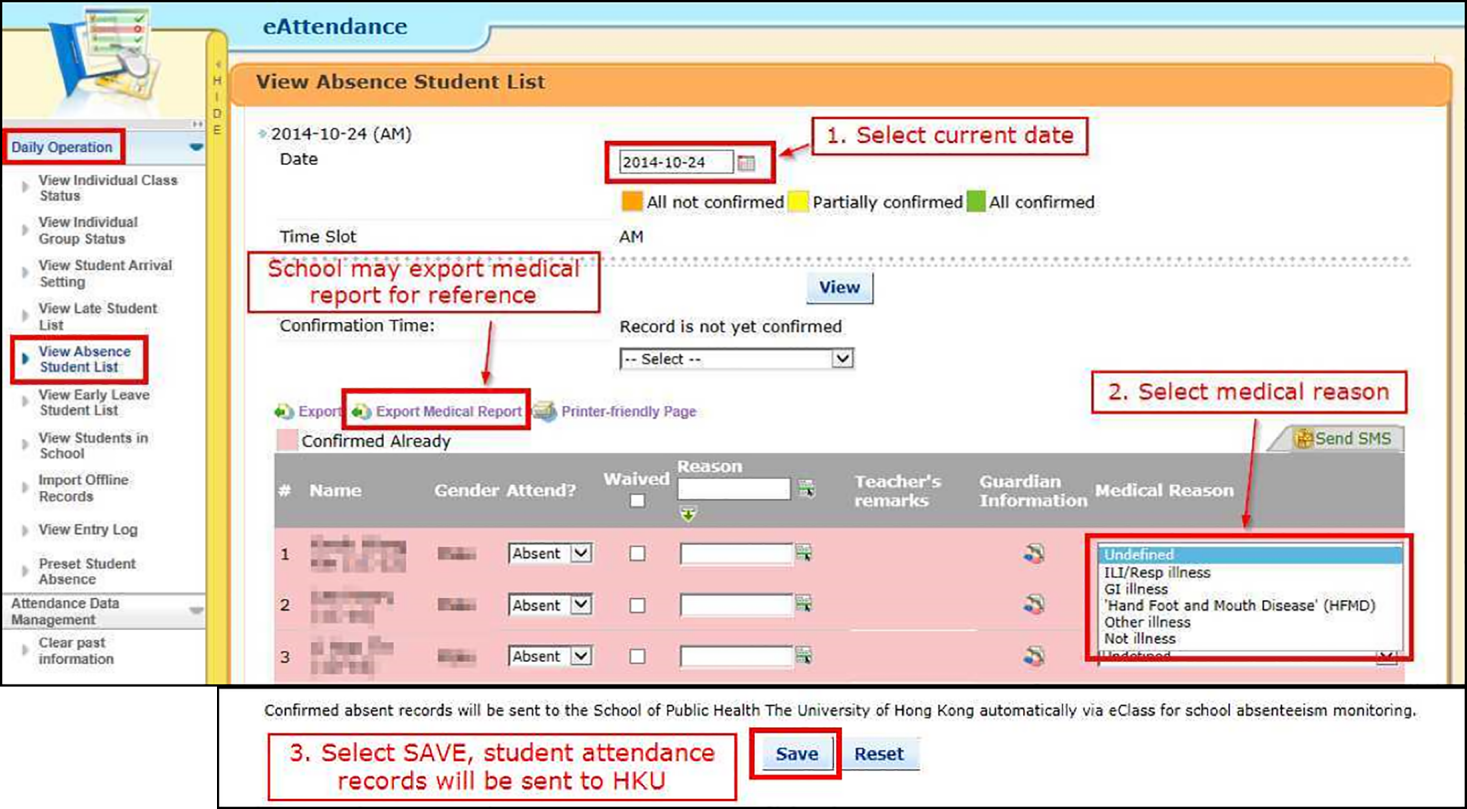
Screenshots of the user interface on the eClass eAttendance module, showing the steps for easy and standardised reporting of specific cause of absence using the drop-down menu (step 2) and for automatic submission of aggregated surveillance data in a standardised format by the Save button (step 3).

Attendance data in participating schools were automatically registered when students swiped their smart student identity card on the card reader on entering their school (Figure 2). Absenteeism was defined as a full day absence where the swiping of a smart card was never registered during the whole school day. Partial days of absenteeism, such as lateness to school with late card swiping or early departure, were not counted. On a daily basis, a list of absent students would be displayed automatically on the user interface of the system in each school for easy checking. In some participating schools, the specific causes of absence were then individually ascertained by telephone call and manually recorded into the system in free text format by designated school staff. In our updated surveillance system, a simple drop-down menu was added to the user interface (Figure 3) to allow for easy and standardized reporting of common causes of absence, such as ILI; gastroenteritis; hand, foot and mouth disease; acute conjunctivitis; other illnesses with specification; or non-sickness-related absence, to facilitate the capturing of these more specific data.

**Figure 4:**
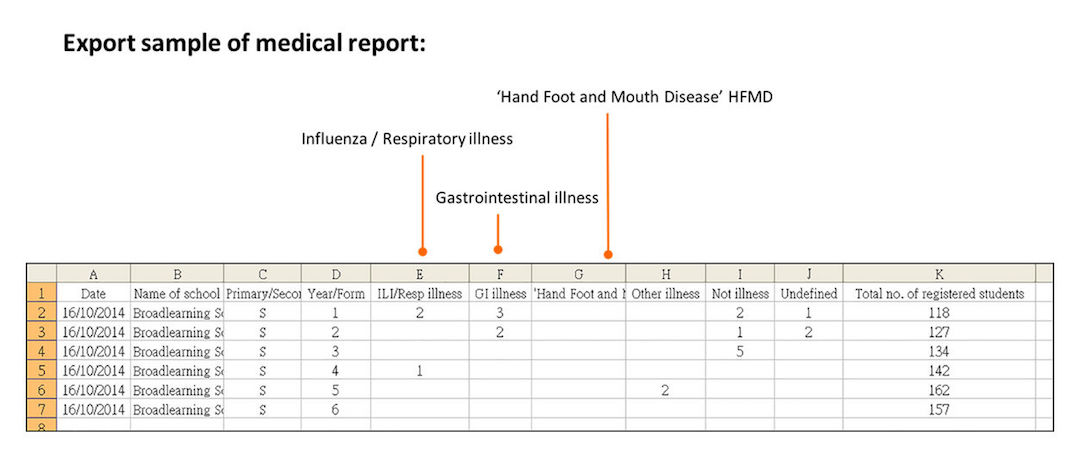
A sample of the daily data submission files in a standard comma separated values (CSV) data file format, containing the number of absentees (all-cause and cause-specific) and total number of enrolled students in each form of the school.

### Data Submission

During the initial pilot study (2008-2010), anonymized and aggregated data from participating schools were sent to an email account of our team, which was then manually downloaded from the email account and uploaded to the HKU Web server via Secure Shell. In this surveillance system, a simple *Save* icon was added to the user interface of the school system to facilitate automatic and efficient data submission on a daily basis with minimal workload implication to school (Figure 3). On clicking this icon by the school staff in each participating school, anonymized and aggregated daily absenteeism data would be automatically extracted from the school’s system and sent directly to our central surveillance server via hypertext transfer protocol secure performed via the secure socket layer (SSL) connections with 2048-bit key. Daily data submission files were in a standard comma separated values data file format, containing the number of absentees (all-cause and ILI-specific) and total number of enrolled students in the school (Figure 4).

### Data Flow Architecture and Privacy

For the back end electronic system we constructed for automatic data handling, there are three layers in the data flow architecture, namely (1) Internet, (2) internal network, and (3) restricted network (Figure 1). Internal network and restricted network are protected behind both the external firewall and internal firewall, where both are hosted by HKU. On the Internet, BroadLearning (BL) eClass server collects submitted aggregated school absenteeism records from the participating schools, which were immediately transferred to our server. The BL server and the HKU workstation are the only 2 computers with access allowed by the external firewall and the HKU Web server, with access from any other sources being denied for maximal data security. All transactions on the database server are logged in a separate table in the same database. All access activities on both the HKU Web server and the database server are logged in two sets of files in different folders with World Wide Web Consortium, or W3C, common log format.

### Data Cleaning and Aggregation

After receiving the updated records, we set up automatic processes on our HKU database server for prespecified data processing, cleaning, and aggregation steps to generate the data for further analysis. Automatic data validation steps include checking the school identity, school name, the date of cases, the number of cases, and the number of students on that date. Valid records are transferred in another SSL and inserted into the database on the HKU database server. Invalid records are stored in the HKU Web server folder for further manual checks by the HKU staff.

### Data Analysis

“All-cause absenteeism” was defined as any episode of absence from school for any reasons. “ILI-specific absenteeism” was defined as any episode of absence from school that was labeled as due to either ILI, influenza or upper respiratory tract infection by a doctor or a parent, or when any symptom, including fever or cough or sputum, was mentioned as a cause of the absence if no such diagnostic label was available. Daily numbers of all-cause and ILI-specific absentees were correspondingly aggregated into a weekly count for the whole territory. The weekly absenteeism rate was then calculated by dividing the aggregated weekly absence counts by the total number of enrolled students in all participating schools. This was done for both all-cause and ILI-specific absenteeism. Two streams of reference data, including the proportion of ILI consultations among general outpatient attendance in the existing sentinel GP surveillance network [19], and positive influenza detection rate from hospital laboratory samples were used for comparison. Timeliness of influenza peak comparison was performed by comparing the weeks when the test data and reference data streams showed the highest activities. Cross-correlation coefficient (CCC) analyses using Pearson product-moment correlation were also done to assess the maximal correlation between different time lags of the corresponding test and reference data. All data received were anonymized for analysis.

**Figure 5:**
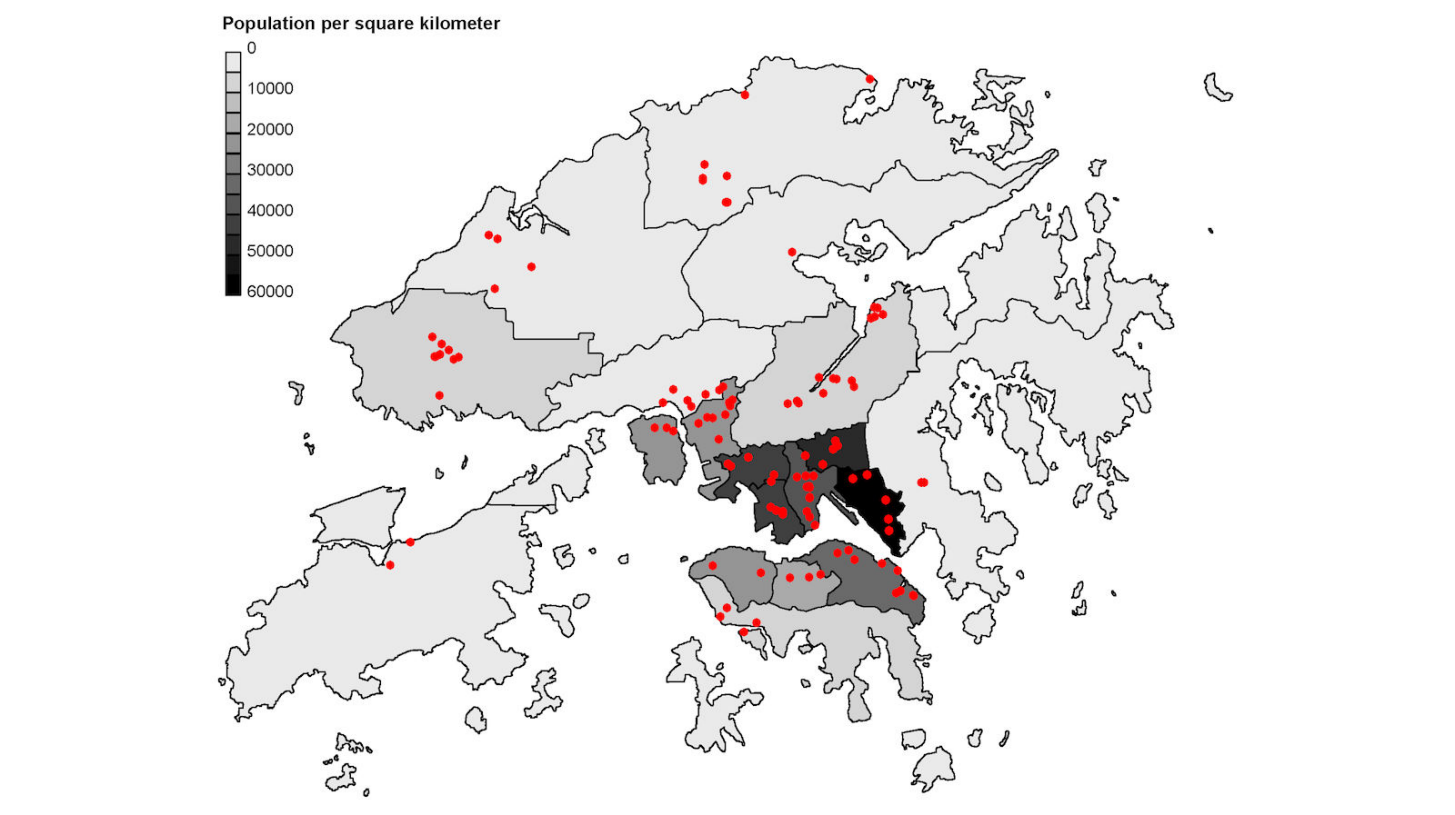
Distribution of participating schools and population density (population/ km2) of the 18 districts in Hong Kong.

### Feedback of Surveillance Intelligence

Two automated processes were developed for the feedback of intelligence from this surveillance system to participating schools and the wider community. A regular surveillance report was disseminated to all participating schools in an electronic format, which consisted of an updated absenteeism trend in the community and for each participating school, together with an interpretation of the overall influenza disease activity in the community and additional value-added health advice based on the current disease activity. We also included the data stream in a surveillance dashboard that we previously developed for dissemination of surveillance intelligence to the community [21].

### Feasibility of Routine Surveillance

We successfully recruited a total of 107 schools (including 66 primary schools and 41 secondary schools) from all 18 districts in Hong Kong to participate in the project (Figure 5), with a total of 75,052 enrolled students, covering 9.83% (107/1088) of schools, 11.6% (66/569) of primary schools and 7.9% (41/519) of secondary schools, and 10.19% (75,052/736,229) of the student population in Hong Kong. All participating schools received a system update of their *eClass* system, with a training session offered to responsible staffs for operating the updated system interface. Besides data submission using the updated system interface as detailed above, no additional workload was imposed on school staffs. A telephone hotline was also maintained by our staff to answer any queries and offer technical assistance that may be needed. With the newly developed system, daily absenteeism data from all participating schools were collected for 2 academic years from September 2012 to June 2014; each academic year starts in September and finishes in June of the subsequent year in Hong Kong, covering a total of four seasonal influenza epidemics locally (two winter and two summer epidemics). In general, the system took around 3 to 4 working days from data capturing to information dissemination. The longest time lag was the lag of 2 to 3 days between data submission and completion of data analysis, mainly to wait for data from all schools to be submitted. Reporting of all-cause absenteeism data was complete (100%) by all 107 participating schools reported. The number of schools reporting ILI-specific absenteeism data using the drop-down menu was initially only 13 (12.1%, 13/107) when they were first recruited, which increased to 38 (35.5%, 38/107) by the end of the project.

**Figure 6:**
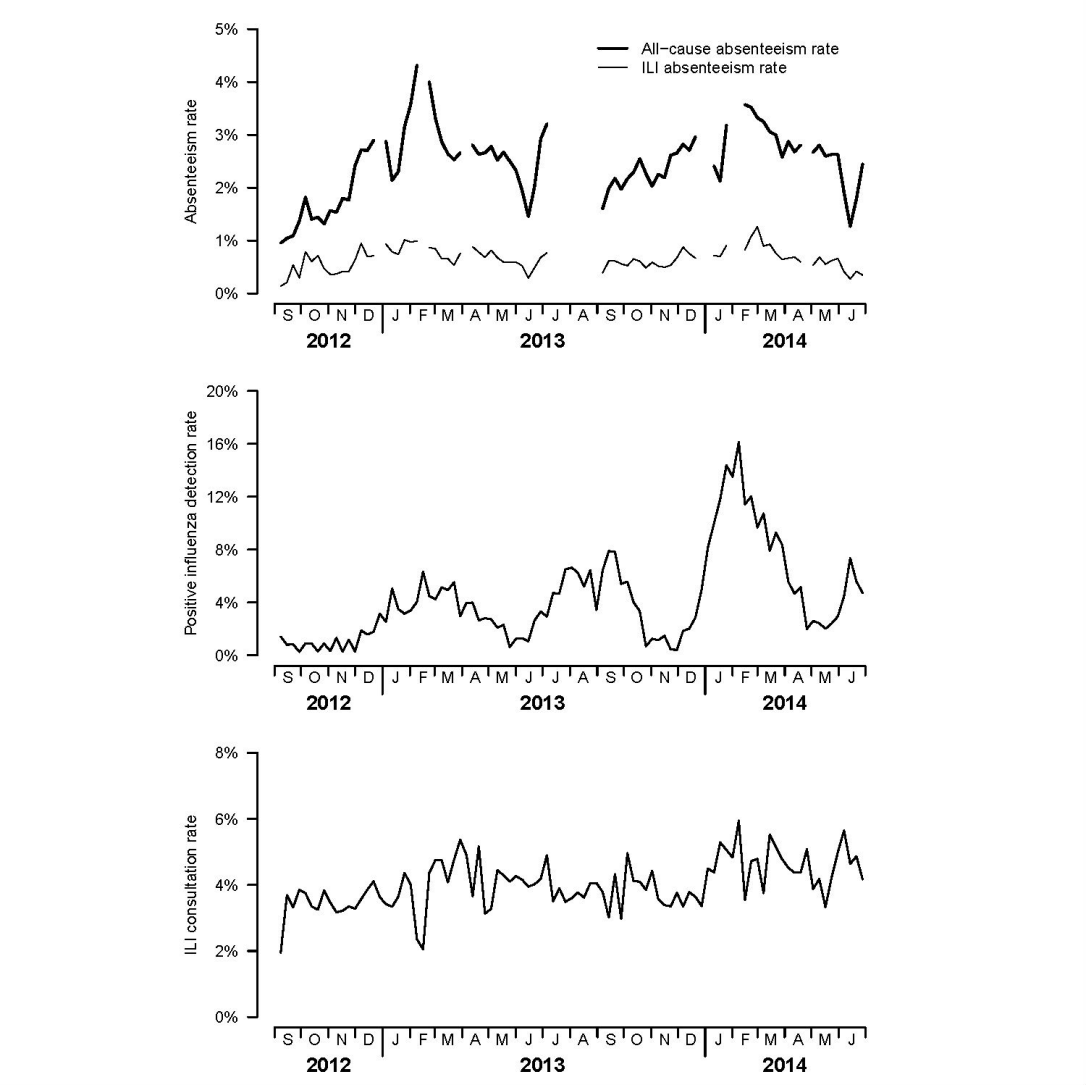
All-cause (thick line) and influenza-like illness (ILI)-specific absenteeism rate (thin line) of students from participating local schools (top), positive influenza isolation rate from Queen Mary Hospital, Hong Kong (middle), and ILI consultation rate of the sentinel general practitioner (GP) network (bottom), 2012-2014.

Among all 75,052 students in 107 schools, there were a mean all-cause weekly absence rate of 2.5% (ranging from 0%-6.4%) and mean ILI-related daily absence rate of 0.7% (ranging from 0%-1.6%) over the study period. Figure 6 shows the temporal patterns of all-cause and ILI-specific school absenteeism rates in relation to two other sources of surveillance data, including the proportion of ILI patients among general outpatient consultations in the existing sentinel GP surveillance network [19] and positive influenza detection rate from hospital samples. Generally, the rate of school absenteeism varied over the course of 2 years, with four periods of elevated absence over the study period that corresponded well with seasonal influenza activity as reflected by the laboratory detection data. Compared with data from the sentinel GP network, the rise, peak, and fall of influenza epidemic activities were much better delineated by the temporal trends reflected by either the all-cause or ILI-specific absenteeism rate. Two of the four epidemic peaks matched exactly temporally with the GP data. The remaining two peaks (early 2013 and early 2014), shown by the school-based surveillance, occurred 1 to 2 weeks before the peaks shown by the GP data. Result of cross-correlation analysis revealed that the maximum correlation occurred with a 3-week lag with the test data preceding the reference data, both between the ILI-specific absenteeism and laboratory data (Max CCC=.434 at lag=3) and between the ILI specific absenteeism and the GP data (Max CCC=.311 at lag=3). These observations agreed with our previous finding from the pilot study that epidemic peaks demonstrated by the school-based surveillance system can precede those shown by the sentinel GP network by 1 to 3 weeks [17].

Feedback from participating schools suggested that the surveillance system was generally well accepted, with 47/72 schools responding to our survey performed when the system had been running for 2 years. We found that 81% of schools agreed that the upgraded system was simple and easy to operate, and 64% of schools responded that data from the system were useful in enhancing their understanding of ILI activity in their schools.

## Discussion

### Principal Findings

We have reported the implementation of a comprehensive ILI surveillance system in schools to fill an existing surveillance gap in Hong Kong. Our study demonstrated the feasibility of using smart card–based technology for tracking attendance data, with the automation of subsequent steps by additional back end systems, for infectious disease surveillance in primary and secondary schools settings. As far as we are aware, this is the first prospective ILI surveillance system among schools in Hong Kong and the first disease surveillance system utilizing smart card technology in the world. Our system is designed to support the various functions of an information infrastructure needed to support pandemic response as detailed in the PROSPER protocol. The continuous baseline surveillance data collected in a stable and standardized manner in interpandemic times can help to inform the CNA and RDM functions. The customizability of the system helps to calibrate detection algorithms in the CNA function with regard to the new pathogen and epidemic. Data from the system also allows OIA for school closure and other relevant public health interventions targeted to reduce disease transmission in the school setting [18].

Although absenteeism surveillance in schools is a component of influenza surveillance and pandemic preparedness plan of many countries, this is generally neither the primary responsibility nor of a high priority for frontline teachers facing many competing educational and administrative duties in the school setting. For any surveillance system to be sustainable, additional workload for system operation needs to be minimized to enhance its acceptability and stability. Different Web-based approaches to facilitate data reporting were tried in previous disease surveillance studies, including a short-term system built for special events [22] and an adjunct system during the 2009 evolving pandemic [11]. Workload minimization, however, was not always achievable in practice, especially when an unfamiliar approach was being introduced in a new system. A recent school absenteeism surveillance system employing mobile devices and Web-based platforms for data reporting had poor acceptability and sustainability as a result of the extra workload required outside the normal daily routine of teachers [15]. Later attempts to use fingerprint scanning for automatic attendance data capture were also not successful as a result of the technical difficulty for a significant number of students to be accurately scanned by the system [15].

Comparing with other electronic approaches, smart card–based technology represents a simple and more reliable approach for automatic data capture. Its capabilities for automatic data capture and immediate compilation for downstream uses offer an advantage of improved data timeliness, making it an ideal approach to inform real-time disease surveillance with minimal additional workload implication for its ongoing collection. By riding on an existing electronic school administration system routinely in use at schools and already familiar to the frontline user, our system avoided potential problems with acceptability and helped to minimize additional workload required for data collection and system operation.

Our design approach may serve as an example to demonstrate how starting from an existing platform with some very simple system customization may help to facilitate the implementation and improve the functioning of a surveillance system. The addition of a drop-down menu is aimed to reduce the workload for the reporting of disease-specific information. This may have contributed to improve acceptability of the procedure as demonstrated by the increasing number of schools willing to collect and report such data over the course of the study. The standardized reporting of disease-specific absenteeism data is aimed to improve specificity of the system and offered a theoretical potential for the system to adapt for surveillance of other infectious diseases of outbreak potential in the school setting. Although we currently analyzed our data in weekly aggregation as all other existing local ILI surveillance systems to facilitate comparison, data from our system may allow for daily analysis when a more frequent surveillance is deemed necessary during an emerging epidemic.

### Limitations

Our system has a number of limitations. First, validity of school absenteeism data in reflecting influenza activity is contingent on the compliance of students refraining from going to schools when being sick. However, we cannot ascertain the degree of nonadherence of schools and students to this policy, which may undermine the performance of this system. This may also affect the generalizability of the results to places where illness absenteeism practice are different because of local cultural or socioeconomic factors. Second, the ascertainment of the cause of absence still has to be done though a manual process. As a result of the additional workload implication, cause-specific absence data were only captured by a portion of schools in our system. This could be mitigated in future by enhancing the system to collect information from parents, for example, by sending an email or SMS text messaging (short service message, SMS) to parents to notify them of their child’s absence and requesting them to provide a reason, with options such as ILI given. Third, data collection is only feasible on normal school days, and data gaps are present during school breaks and holidays (Figure 6), which limited the possibility for continuous surveillance during those periods.

### Conclusions

In conclusion, we have demonstrated the feasibility of building and implementing a large-scale surveillance system by employing a routinely adopted smart card–based approach for automatic data capture in schools, supplemented by simple automation of later steps required for prospective surveillance. The absenteeism data we collected from the system reflected ILI activity in the community and we were able to detect an upsurge in its disease activity in a more timely manner than other existing surveillance data. As we previously demonstrated that the alerting of the onset of an epidemic can be greatly enhanced in terms of sensitivity and timeliness by the monitoring of multistream surveillance data [23], our absenteeism data is potentially useful for supplementing existing systems in Hong Kong for monitoring the trend of influenza disease activity in the community. With the increasing popularity of smart card–based electronic health record systems in different health care utilization settings [24,25], it offers an unprecedented potential for novel surveillance and risk monitoring systems to be developed based on this technology [26]. Our experience would be referential for other countries and cities facing a similar problem and in need of improving their existing surveillance systems, particularly in showing how this can be achieved by suitably and creatively adapting to an existing system.

## Abbreviations

BL: BroadLearning
CCC: cross-correlation coefficient
CNA: capacity and needs analysis
CHP: Centre for Health Protection
GP: general practitioner
HKU: The University of Hong Kong
ILI: influenza-like illness
OIA: outcome and impact assessment
PROSPER: Protocol for a Standardized information infrastructure for Pandemic and Emerging infectious disease Response
RDM: response design modeling
SSL: secure socket layer

## References

[1] Glezen WP, Couch RB (1978). Interpandemic influenza in the Houston area, 1974-76. N Engl J Med.

[2] Longini Jr IM, Koopman JS, Monto AS, Fox JP (1982). Estimating household and community transmission parameters for influenza. Am J Epidemiol.

[3] Neuzil KM, Hohlbein C, Zhu Y (2002). Illness among schoolchildren during influenza season: effect on school absenteeism, parental absenteeism from work, and secondary illness in families. Arch Pediatr Adolesc Med.

[4] Cauchemez S, Ferguson NM, Wachtel C, Tegnell A, Saour G, Duncan B, Nicoll A (2009). Closure of schools during an influenza pandemic. Lancet Infect Dis.

[5] Mikolajczyk RT, Akmatov MK, Rastin S, Kretzschmar M (2008). Social contacts of school children and the transmission of respiratory-spread pathogens. Epidemiol Infect.

[6] (2014). Canada.ca.

[7] Mandil A, Bresee J, Tageldin MA, Azad TM, Khan W (2016). Research agenda on persistent and unpredictable threat of influenza and emerging respiratory infections: a public health necessity in the Eastern Mediterranean Region. East Mediterr Health J.

[8] Suzue T, Hoshikawa Y, Nishihara S, Fujikawa A, Miyatake N, Sakano N, Yoda T, Yoshioka A, Hirao T (2012). The new school absentees reporting system for pandemic influenza A/H1N1 2009 infection in Japan. PLoS One.

[9] Takahashi H, Fujii H, Shindo N, Taniguchi K (2001). Evaluation of the Japanese school health surveillance system for influenza. Jpn J Infect Dis.

[10] Kightlinger L, Horan V (2013). School illness absenteeism during 2009 influenza A (H1N1) pandemic--South Dakota, 2009-2010. S D Med.

[11] Kom Mogto CA, De Serres G, Douville Fradet M, Lebel G, Toutant S, Gilca R, Ouakki M, Janjua NZ, Skowronski DM (2012). School absenteeism as an adjunct surveillance indicator: experience during the second wave of the 2009 H1N1 pandemic in Quebec, Canada. PLoS One.

[12] Schmidt WP, Pebody R, Mangtani P (2010). School absence data for influenza surveillance: a pilot study in the United Kingdom. Euro Surveill.

[13] Besculides M, Heffernan R, Mostashari F, Weiss D (2005). Evaluation of school absenteeism data for early outbreak detection, New York City. BMC Public Health.

[14] Dailey L, Watkins RE, Plant AJ (2007). Timeliness of data sources used for influenza surveillance. J Am Med Inform Assoc.

[15] Lawpoolsri S, Khamsiriwatchara A, Liulark W, Taweeseneepitch K, Sangvichean A, Thongprarong W, Kaewkungwal J, Singhasivanon P (2014). Real-time monitoring of school absenteeism to enhance disease surveillance: a pilot study of a mobile electronic reporting system. JMIR Mhealth Uhealth.

[16] Hendry M (2007). Multi-application Smart Cards: Technology and Applications.

[17] Cheng CK, Cowling BJ, Lau EH, Ho LM, Leung GM, Ip DK (2012). Electronic school absenteeism monitoring and influenza surveillance, Hong Kong. Emerg Infect Dis.

[18] Timpka T, Eriksson H, Gursky EA, Strömgren M, Holm E, Ekberg J, Eriksson O, Grimvall A, Valter L, Nyce JM (2011). Requirements and design of the PROSPER protocol for implementation of information infrastructures supporting pandemic response: a nominal group study. PLoS One.

[19] Centre for Health Protection, Department of Health (2014). The Government of Hong Kong Special Administrative Region.

[20] Centre for Health Protection, Department of Health (2014). The Government of Hong Kong Special Administrative Region.

[21] Cheng CK, Ip DK, Cowling BJ, Ho LM, Leung GM, Lau EH (2011). Digital dashboard design using multiple data streams for disease surveillance with influenza surveillance as an example. J Med Internet Res.

[22] Sugiura H, Ohkusa Y, Akahane M, Sugahara T, Okabe N, Imamura T (2010). Construction of syndromic surveillance using a web-based daily questionnaire for health and its application at the G8 Hokkaido Toyako Summit meeting. Epidemiol Infect.

[23] Lau EH, Cowling BJ, Ho LM, Leung GM (2008). Optimizing use of multistream influenza sentinel surveillance data. Emerg Infect Dis.

[24] Tsai WH, Kuo HC (2007). The Internet and healthcare in Taiwan: value-added applications on the medical network in the National Health Insurance smart card system. Int J Electron Healthc.

[25] Lambrinoudakis C, Gritzalis S (2000). Managing medical and insurance information through a smart-card-based information system. J Med Syst.

[26] Mercuri M, Rehani MM, Einstein AJ (2012). Tracking patient radiation exposure: challenges to integrating nuclear medicine with other modalities. J Nucl Cardiol.

